# Myxofibrosarcoma of the leg: A diagnostic challenge

**DOI:** 10.1002/ccr3.3414

**Published:** 2020-10-21

**Authors:** Imène Rachdi, Fatma Daoud, Fatma Khanchel, Ibrahim Arbaoui, Mehdi Somai, Hana Zoubeidi, Zohra Aydi, Besma Ben Dhaou, Achraf Debbiche, Fatma Boussema

**Affiliations:** ^1^ Internal Medicine Department Habib Thameur Hospital Tunis Tunisia; ^2^ Faculty of Medicine of Tunis University of Tunis el Manar Tunis Tunisia; ^3^ Department of Pathology Habib Thameur Hospital Tunis Tunisia

**Keywords:** diagnosis, leg, myxofibrosarcoma

## Abstract

We should keep in mind slowly growing malignancies when the lesion is located close to the synovial regions of the extremities. The diagnosis of certainty of myxofibrosarcoma is histological and is based on the demonstration of the myxoid matrix, fibroblastic cells with a curvilinear arrangement of the vessels.

## Graphical abstract

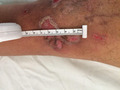

## 1 INTRODUCTION

Myxofibrosarcoma is a common sarcoma of the soft tissue of the extremities in the elderly. The diagnosis of certainty is histological. We report the case of myxofibrosarcoma of the leg occurring in a 68‐year‐old patient.

Sarcomas represent 0.5%‐1% of malignant tumors in adults.^1^ They are a real diagnostic and therapeutic challenge for multidisciplinary medical teams.^2^ To our Knowledge, few cases were reported in Tunisia.^3^ The pathologist must establish the malignant potential of the lesion, its degree of aggressiveness, report the prognostic factors, and evaluate the quality of the treatment.

We report the case of myxofibrosarcoma of the leg occurring in a 68‐year‐old patient.

## 2 OBSERVATION

**FIGURE 1:**
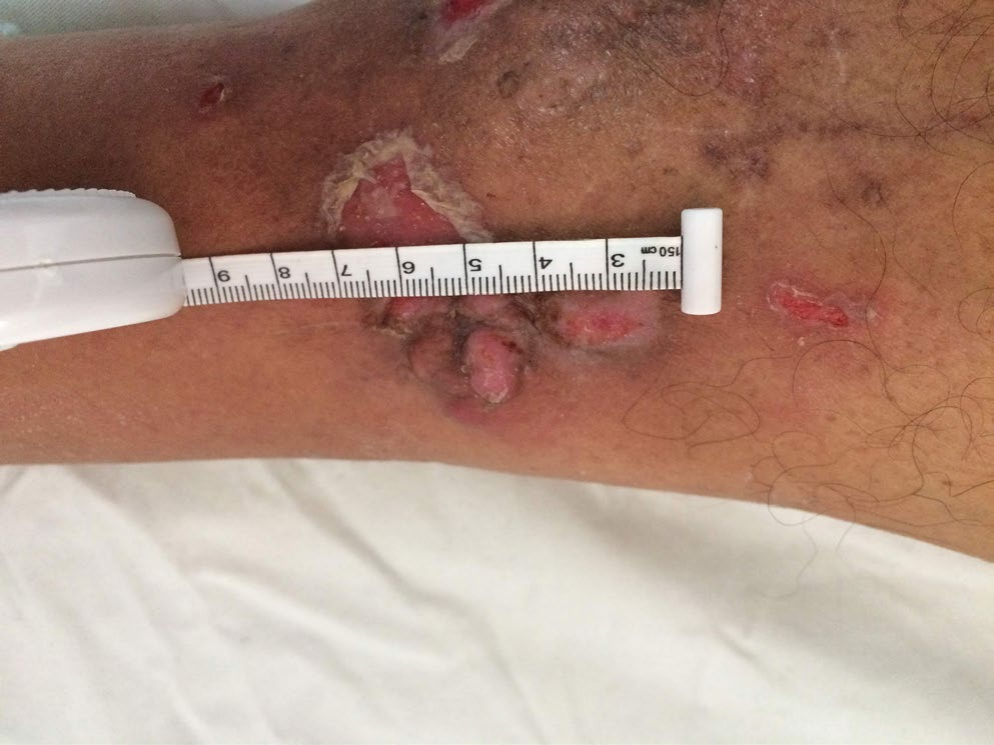
An erythemato‐papular infiltrated lesion on the anterior side of the lower leg and a second hyperpigmented lesion on the anterior‐inner side

**FIGURE 2:**
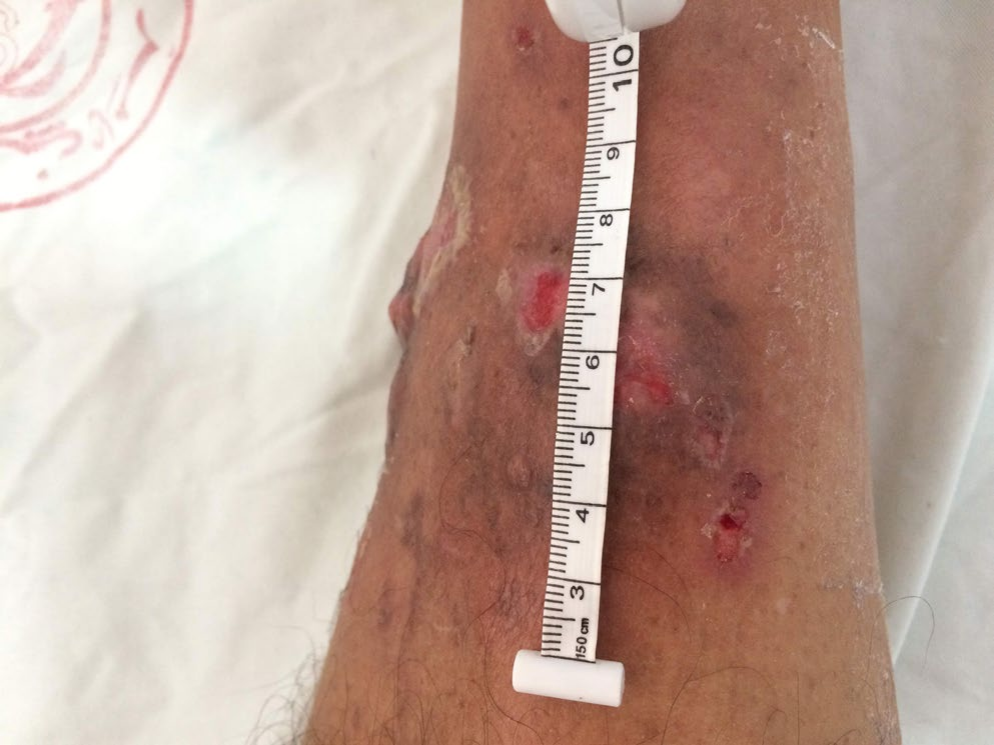
An erythemato‐papular infiltrated lesion on the anterior side of the lower leg and a second hyperpigmented lesion on the anterior‐inner side

A 68‐year‐old male, without any particular history, consulted for a swelling of the right leg. There was no history of trauma. He did not present a fever nor a deterioration of the general state. Physical examination revealed a an erythemato‐papular infiltrated lesion on the anterior side of the lower leg and a second hyperpigmented lesion on the anterior‐inner side of the leg (Figures 1 and 2). The mass was painless without local inflammatory signs or satellite lymphadenopathy.

The biological examinations (Blood count, inflammatory balance, phosphocalcic balance, and protein electrophoresis) were normal.

**FIGURE 3:**
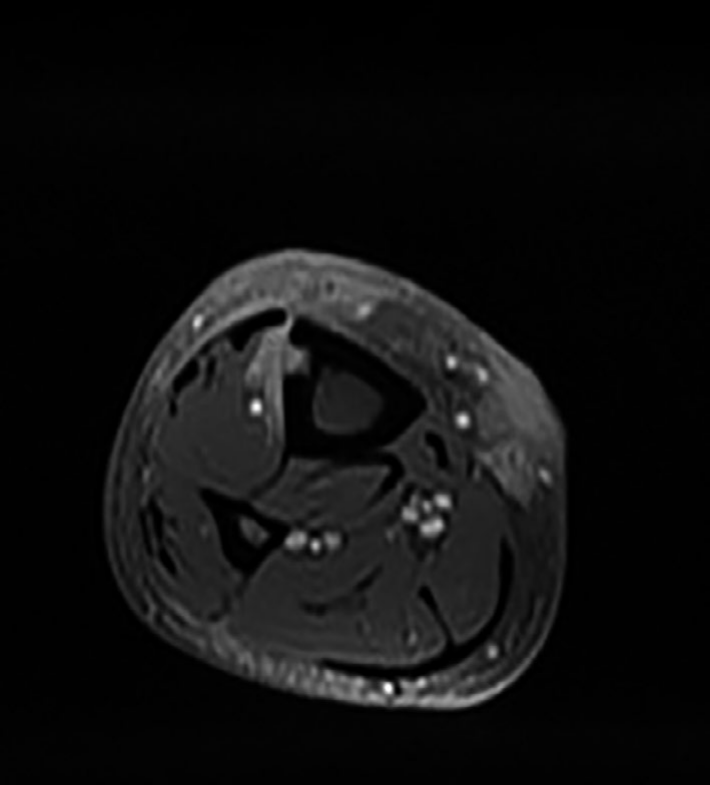
MRI showed a fibrous cutaneous lesion of the anterior surface of the limb lower right leg with trans‐aponeurotic expansion and contiguity corticoperiosteal extension to anterolateral tibia

The X‐ray of the leg showed an osteolytic image by near to the skin lesion with respect to the cortical. MRI showed a fibrous cutaneous lesion of the anterior surface of the limb lower right leg with trans‐aponeurotic expansion and contiguity corticoperiosteal extension to anterolateral tibia. A second lesion was noted in its intramuscular extension of the fibulars coming into contact over about 180 degrees of the medial circumference of the tibial artery (Figure 3).

**FIGURE 4:**
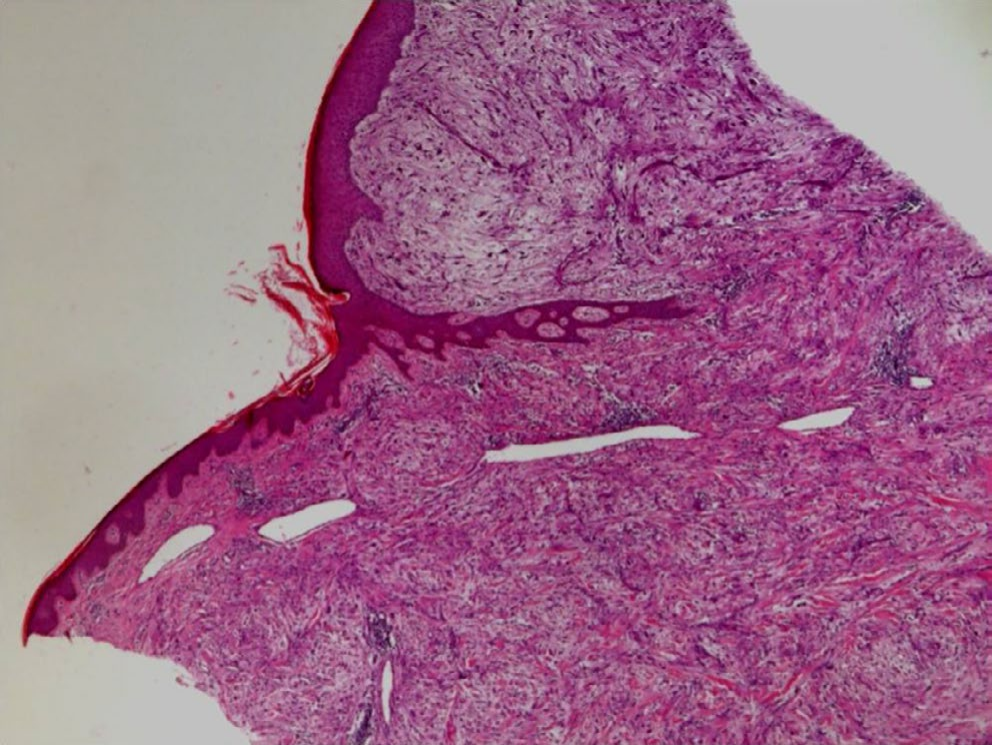
Dermo‐hypodermic mesenchymal proliferation (hemato‐eosin ×100)

**FIGURE 5:**
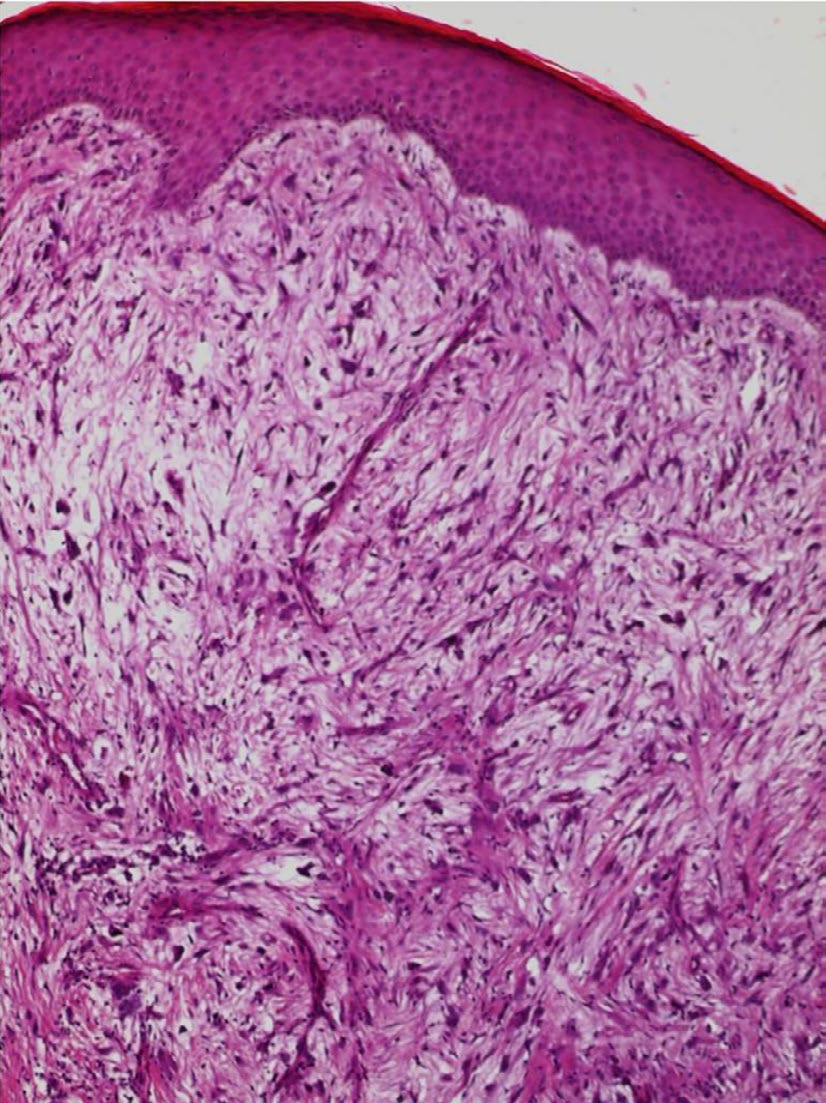
Variable cellularity with a proliferative myxoid stroma (He ×200). In cartridge: cell pleomorphism with mitosis figures (He ×400)

Biopsies of the lesion with surgical removal of the tumor were performed. Histological examination of the piece confirmed the diagnosis of a myxofibrosarcoma whose high grade was justified by hypercellularity, high mitotic activity, pleomorphism, and intralesional necrosis (Figures 4 and 5). The tumor was located deep in relation to the superficial fascia.

Immunohistochemical study was negative. The patient underwent resection of the tumor. After follow‐up of 6 months, he died probably of pulmonary embolism.

## 3 DISCUSSION

Myxofibrosarcoma is one of the most common sarcomas in adults. The term myxofibrosarcoma is currently preferred to the former name of malignant myxoid fibrous histiocytoma. The mean age of onset is 65 years (40‐80 years),^1^ the preferred location is the limbs. In particular, the tumor can be either deep or superficial, cutaneous, or subcutaneous (20%‐70%) of case. The average size is 6 cm (1a 10 cm).^2^ Rare are the cases of fibromyxosarcoma which have been reported in Tunisia. Khelil et al described a case of a 42‐year‐old woman with fibromyxosarcoma in the foot. The diagnosis of sarcoma was suspected by magnetic resonance imaging. Confirmation of fibromyxosarcoma was confirmed by histological study. The treatment was surgical with neo‐adjuvant radiotherapy with good progress.^3^ Histologically, this entity covers a broad morphological spectrum, ranging from myxoma‐like cell‐like to low‐ and high‐grade tumors.^4^

The diagnosis of this condition is based on the characteristics of the myxoid zones. The proportion of myxoid areas required to diagnose myxofibrosarcoma remains controversial.

Some authors require that 50% of tumor volume is myxoid area. For other authors, 10% of myxoid areas are sufficient for diagnosis.^5^ High‐grade myxofibrosarcoma is characterized by hypercellularity, high mitotic activity, pleomorphism, and intralesional necrosis. It is located deep in relation to the superficial fascia.

The differential diagnosis for this lesion is broad. It includes most of the benign and malignant myxoid lesions and can range from an infectious inflammatory process to malignant myxofibrosarcoma.

Fibromyxosarcoma, whose name has evolved over time, is also called “myxoid variant of malignant fibrous histiocytoma.” These nominations constitute a histological description or terminology. This thus makes it possible to underline the differential diagnoses, which are the other sarcomas of the soft tissues. With the introduction of more stringent morphologic and immunohistochemical criteria in 2002 and reaffirmed in 2013, the World Health Organization renamed the myxoid variant of malignant fibrous histiocytoma with a predominant myxoid component (>50%) myxofibrosarcoma.^6^, ^7^

Roland et al reported in a study of 3574 patients the distribution of the different histotypes of soft tissue sarcoma. These different histotypes are the differential diagnoses of fibromyxosarcoma (liposarcoma, undifferentiated pleomorphic sarcoma, leiomyosarcoma, gastrointestinal stromal tumor, synovial sarcoma, and malignant peripheral nerve sheath tumor).^8^

MRI may be contributory to the diagnosis by showing a tumor with T1‐weighted intermediate signal, heterogeneously enhanced and signal heterogeneity in T2.

High‐grade myxoid lesions may appear cystic, with one component nodular non‐cystic revealed after injection of gadolinium.^9^

On x‐ray calcification and osteolysis are visualized in 5%‐20% of case. Local recurrence is observed in 60% of cases, when metastases are observed in 20% in cases in pleomorphic tumors.^10^, ^11^

The treatment is often radical and relies on radical surgical excision, and a complement of neo‐adjuvant chemo‐radiotherapy can be proposed in case of high‐grade tumor.^10^

## 4 CONCLUSION

High‐grade myxofibrosarcomas are poor prognosis. Imaging has a crucial role in diagnosis. Diagnostic confirmation by prior biopsy allows to program a tumor resection in en‐bloc. Management should be multidisciplinary, and sometimes, neo‐adjuvant chemotherapy and radiation therapy are required for locally advanced tumors.

## CONFLICT OF INTEREST

None declared.

## AUTHOR CONTRIBUTIONS

IR and FD: wrote the manuscript with support of IA. MS, HZ, ZA, BBD, and FB: approved the final version of the manuscript. FK and ACD: analyzed pathology images.

## ETHICAL APPROVAL

Not applicable.
